# Frequent use of IGHV3-30-3 in SARS-CoV-2 neutralizing antibody responses

**DOI:** 10.3389/fviro.2023.1128253

**Published:** 2023-03-01

**Authors:** Pradeepa Pushparaj, Andrea Nicoletto, Xaquin Castro Dopico, Daniel J. Sheward, Sungyong Kim, Simon Ekström, Ben Murrell, Martin Corcoran, Gunilla B. Karlsson Hedestam

**Affiliations:** 1 Department of Microbiology, Tumor and Cell Biology, Karolinska Institutet, Stockholm, Sweden; 2 Department of Biomedical Engineering, Lund University, Lund, Sweden

**Keywords:** SARS-CoV-2, immunoglobulin germline genes, allelic diversity, copy number variation, antibodies

## Abstract

The antibody response to SARS-CoV-2 shows biased immunoglobulin heavy chain variable (IGHV) gene usage, allowing definition of genetic signatures for some classes of neutralizing antibodies. We investigated IGHV gene usage frequencies by sorting spike-specific single memory B cells from individuals infected with SARS-CoV-2 early in the pandemic. From two study participants and 703 spikespecific B cells, the most used genes were IGHV1-69, IGHV3-30-3, and IGHV3-30. Here, we focused on the IGHV3-30 group of genes and an IGHV3-30-3-using ultrapotent neutralizing monoclonal antibody, CAB-F52, which displayed broad neutralizing activity also in its germline-reverted form. IGHV3-30-3 is encoded by a region of the IGH locus that is highly variable at both the allelic and structural levels. Using personalized IG genotyping, we found that 4 of 14 study participants lacked the IGHV3-30-3 gene on both chromosomes, raising the question if other, highly similar IGHV genes could substitute for IGHV3-30-3 in persons lacking this gene. In the context of CAB-F52, we found that none of the tested IGHV3-33 alleles, but several IGHV3-30 alleles could substitute for IGHV3-30-3, suggesting functional redundancy between the highly homologous IGHV3-30 and IGHV3-30-3 genes for this antibody.

## Introduction

Since the emergence of SARS-CoV-2 at the end of 2019, research groups around the world have characterized the antibody responses to the virus surface glycoprotein, spike (S), using serological assays and by isolating monoclonal antibodies (mAbs). It is now well-established that the receptor binding domain (RBD) of S is the main target for neutralizing antibodies (1–3), though rare neutralizing antibodies that target the N-terminal domain (NTD) or subunit 2 (S2) of S have also been reported (4–6).

In November 2020, the first variant of concern (VoC), Alpha, emerged. Alpha was followed by a series of VoCs, many of which displayed partial resistance to neutralizing antibodies, so-called immune escape variants. The sub-lineages of Omicron that now contribute to the majority of infections, are considerably more neutralization resistant than the ancestral virus (7–11). The fact that antibody escape variants emerge in populations with a high level of seropositivity, obtained from either infection, vaccination or a combination of both, demonstrates that neutralizing antibodies exert selection pressure on the virus.

The antibody response to S is highly polyclonal and engages many different IGHV genes (12–16). Several studies have reported the over-representation of certain IGHV genes, such as IGHV1-69, IGHV3-30 and IGHV3-53, in SARS-CoV-2 S-binding antibodies compared to in the total antibody repertoire (12, 14–18). Of these, IGHV1-69 and IGHV3-30 are also often used in antibody responses to other antigens as they are frequent in the IgM repertoire, and thus there is a high likelihood that naïve B cells encoding one of these genes will encounter antigen. In contrast, IGHV3-53 is frequently used in the response to SARS-CoV-2 RBD despite being infrequent in the total IgM repertoire, suggesting preferential usage (19). Studies have shown that germline-encoded amino acid motifs in IGHV3-53 make direct contacts with the RBD, explaining the potency of this class of antibodies despite low levels of somatic hypermutation (SHM) (3, 19, 20).

There are 52 known IGHV genes in humans (21, 22), many of which display extensive allelic and copy number variation between individuals (22–25). Thus, the IGHV genotypes and haplotypes across individuals are highly personal and, together with the IGHD, IGHJ and the light chain genes, shape the composition of the expressed IgM repertoire in each individual.

The IGHV region encompassing IGHV3-30, IGHV3-30-3, IGHV3-30-5 and IGHV3-33 are particularly variable between individuals. These genes are highly similar at the nucleotide level, consistent with a recent evolutionary origin due to gene duplication events. IGHV3-33 is more distantly related at the nucleotide level but differs from IGHV3-30 and IGHV3-30-3 by only one or two amino acids. These genes, often referred to as the IGHV3-30 group of genes, are some of the most highly used IGHV genes in SARS-CoV-2 neutralizing antibodies (15, 16, 18). Several antibodies that use these genes bind and neutralize the virus even in their germline state, suggesting a role for the germline-encoded residues in epitope binding (17). The frequent usage of these closely related yet polymorphic genes in the antibody response to SARS-CoV-2 raises the question of how this germline-encoded diversity influences antigen binding properties of antibodies.

Here, we studied of a cohort of healthcare workers who were infected with SARS-CoV-2 during spring 2020, from which we recently reported the isolation of a set of mAbs including the ultrapotent mAb, CAB-F52, which used IGHV3-30-3 (16). Here, we characterized this mAb further by reverting the mAb heavy chain (HC) to the germline sequence to elucidate the role of SHM for the neutralizing activity. We also investigated the presence of IGHV3-30 family genes and alleles in this cohort and in an additional cohort of healthy individuals to better understand the germline-encoded variation in the region around IGHV3-30-3. We observed a high allelic and copy number variation of these genes with many individuals lacking the IGHV3-30-3 gene on both chromosomes. Finally, we performed IGHV-region swaps to ask if IGHV3-30 or IGHV3-33 can substitute for IGHV3-30-3 in CAB-F52, and thus potentially compensate for the frequently observed homozygous absence of IGHV3-30-3 in the neutralizing Ab response to SARS-CoV-2.

## Materials and methods

### Sample collection

All data were collected as previously described in Pushparaj et al. (16). In brief, healthcare staff at the Karolinska University Hospital in Stockholm, Sweden, were invited to take part in an antibody characterization study following a SARS-CoV-2 infection. PCR-positive participants that were infected in May 2020 provided blood samples in December 2020. Informed consent was obtained from all the study participants as part of ethics approvals (Decisions# 2020-01620, 2020-02881 and 2020-05630) from the National Ethical Review Agency of Sweden.

### Cell culture

HEK293F cells, HEK293T cells (ATCC CRL-3216), and human ACE-2 expressing HEK293T-ACE2 cells were cultured in Dulbecco’s Modified Eagle Medium (glucose, with sodium pyruvate) containing with 10% FBS, 100 U/ml penicillin, and 100 μg/ml streptomycin. Cells were maintained in a humidified incubator under culture conditions of 5% CO_2_ at 37°C. Of these, HEK293F cells were used for mAb expression while the others were used for pseudo virus neutralization assay.

### Cloning of germline-reverted mAbs

Using Gibson assembly mixture, germline reverted HC VDJ sequences were cloned into expression vectors with the human constant regions IgG1 (26). Briefly, VDJ germline sequences were designed and synthesized with overhangs matching the ends of the linearized vectors. The Gibson reaction master mix (New England Biolabs) consisted of 50 ng of vector, 30 ng of insert in a 20 μl reaction. This reaction mixture was then incubated at 50°C for 1 hour and transformed into XL10-Gold ultracompetent cells according to the manufacturer’s protocol (Agilent Technologies). Colonies were screened and the positive ones were identified by Sanger sequencing (Genewiz) and expanded using plasmid Midi-prep kit (Qiagen) according to the manufacturer’s instructions.

### Expression and purification of germline-reverted mAbs

Germline-reverted CAB-F52 was generated by co-transfecting the germline HCs with the mature CAB-F52 LC. Antibody expression was performed by co-transfecting 15 μg of each HC and LC plasmid using 30 ml of FreeStyle 293-F cells at a concentration of 1 million cells/ml (≥ 90% viability) maintained in FreeStyle 293-F medium (Life Technologies) with 30μl of Max reagent (Life Technologies). Purification of antibodies was performed 7 days post-transfection using Protein G Sepharose columns (GE Healthcare) and purity was confirmed by SDS-PAGE.

### Preparation of Fab fragments

Fab fragments were obtained by IgG digestion using immobilized papain (Pierce Fab Preparation Kit, ThermoFisher Scientific) according to the manufacturer’s instructions.

### Pseudovirus neutralization assay

Expression vectors encoding the spike proteins of various SARS-CoV-2 strains, including B.1 (D614G), B.1.1.7 (Alpha), B.1.351 (Beta), P.1 (Gamma), B.1.617.2 (Delta), and B.1.621 (Mu) were obtained from the G2P-UK National Virology consortium and Barclay Lab (Imperial College London, UK). B.1.1.529 spike (Omicron) strain was also included and was obtained by molecular cloning from an infected individual as previously (10). To generate pseudovirus particles, the spike-encoding plasmid, a HIV gag-pol packaging plasmid (Addgene #8455), and a lentiviral transfer plasmid encoding firefly luciferase (Addgene #170674) were co-transfected into HEK293T cells using polyethylenimine. The assays were performed by incubating pseudoviruses with serially diluted mAbs for 60 minutes at 37°C, then adding 10,000 HEK293T-ACE2 cells to each well. The cells were incubated for 44-48 hours, and the neutralizing activity of the mAbs was determined by measuring luminescence using Bright-Glo (Promega) on a GloMax Navigator Luminometer (Promega). The neutralizing activity was calculated relative to the average of 8 control wells infected in the absence of serum.

### Hydrogen-deuterium exchange mass spectrometry

The experimental setup consisted of a LEAP H/D-X PAL™ platform, used for automated sample preparation, interfaced to an LC-MS system, comprising an Ultimate 3000 micro-LC coupled to an Orbitrap Q Exactive Plus MS. HDX was performed on RBD with and without Fab obtained from the digestion of CAB-F52 in 10 mM PBS. Apo state (unbound RBD) and epitope mapping (RBD bound to either one of the Fabs) samples were incubated for t = 0, 30, 300, 3000 at 20°C in PBS or HDX labelling buffer of the same composition prepared in D_2_O. The analysis was performed in a single, continuous run and 3 replicates were run for each state and timepoint. Quenching of the labelling reaction was achieved by dilution with 1% TFA, 0.4 M TCEP, 4 M urea, at 1°C. The quenched sample was directly injected and subjected to online pepsin digestion at 4°C. A flow of 50 μL/min 0.1% formic acid was applied for 4 minutes for online digestion and trapping of the samples. Digestion products were subjected to online solid phase extraction and washing with 0.1% FA for 60s on a PepMap300 C18 trap column, which was switched in-line with a reversed-phase analytical column, Hypersil GOLD. Separation was performed at 1°C, with mobile phases of 0.1% formic acid (A) and 95% acetonitrile/0.1% formic acid (B), using a gradient of 5-50% B over 8 minutes and then from 50 to 90% B for 5 minutes. Separated peptides were analyzed on a Q Exactive Plus MS, equipped with a heated electrospray source (HESI) operated at a capillary temperature of 250°C with sheath gas 12 au, auxiliary gas 2 au, and sweep gas 1 au. For HDX analysis MS full scan spectra were acquired at 70000 resolution, automatic gain control (AGC) 3e6, Max ion injection time 200ms and scan range 300-2000 m/z. For identification of generated peptides separate undeuterated samples were analyzed using data dependent MS/MS.

### HDX analysis

A library of peptides containing peptide sequence, charge state and retention time was generated for the HDX analysis by running pepsin-digested, undeuterated samples against the RBD sequence on PEAKS Studio X (Bioinformatics Solutions Inc.). HDX data analysis and visualization were performed using HDExaminer v3.1.1 (Sierra Analytics Inc.). Bound states were analysed in comparison to apo states, using a single charge state per peptide. This analysis allowed only for EX2 kinetics, and the assumption was made that the two first residues of a peptide were unable to hold deuteration. Due to the comparative nature of the measurements, the deuterium incorporation levels for the peptic peptides were derived from the observed relative mass difference between the deuterated and non-deuterated peptides without back-exchange correction using a fully deuterated sample (27). The spectra for all time points were manually inspected; low scoring peptides, obvious outliers and peptides for which retention time correction could not be made consistent were removed. The HDX-MS experiments presented here are reported in accordance with established recommendations (28). A summary of the HDX experimental detail can be found in Supplementary Table 1. In addition, the mass spectrometry raw data and HDExaminer analysis files have been deposited to the ProteomeXchange Consortium *via* the PRIDE partner repository (29) with the dataset identifier PXD031945.

### Analysis of IgM libraries from Gidoni et al

Individual VDJ databases were produced using IgDiscover v0.12.4.dev22+g0bc3365 with the IMGT release 202209-1 (28 February 2022) as the starting database from bulk IgM sequences reported in Gidoni et al. (24).

### Quantification and statistical analysis

Neutralizing IC_50_ values were calculated in Prism v9 (GraphPad Software) by fitting a four-parameter logistic curve, to neutralization by serial 3-fold dilutions. Neutralization was bounded between 0 and 100%. KD and IC50 correlations were determined using Pearson’s R (GraphPad Software). To determine the statistical significance between the groups in the bar plot showing combined IGHV3-30/IGHV3-30-3 frequencies in IgM libraries, Wilcoxon rank sum Q1 exact test was used and the p value was determined to be 0.002.

### Data availability

All data were collected as previously described (16). The HC and LC sequences of CAB-F52 and the IgM repertoire data from the 14 donors are available at GenBank under the accession numbers ON086926 and ON086941. Repertoire data is available from SciLifeLab at: http://doi.org/10.17044/scilifelab.19317512. The HDX data have been deposited to the ProteomeXchange Consortium *via* the PRIDE (29) partner repository with the dataset identifier PXD031945.

### Software availability

The IgDiscover software can be found at http://docs.igdiscover.se/en/stable/. The plot allele module and the rep-seq analysis tools can be found under IgDiscover v0.12.4.dev22+g0bc3365.

## Results

### SARS-CoV-2 spike-specific memory B cell sequencing revealed preferential IGHV gene usage

**Figure 1:**
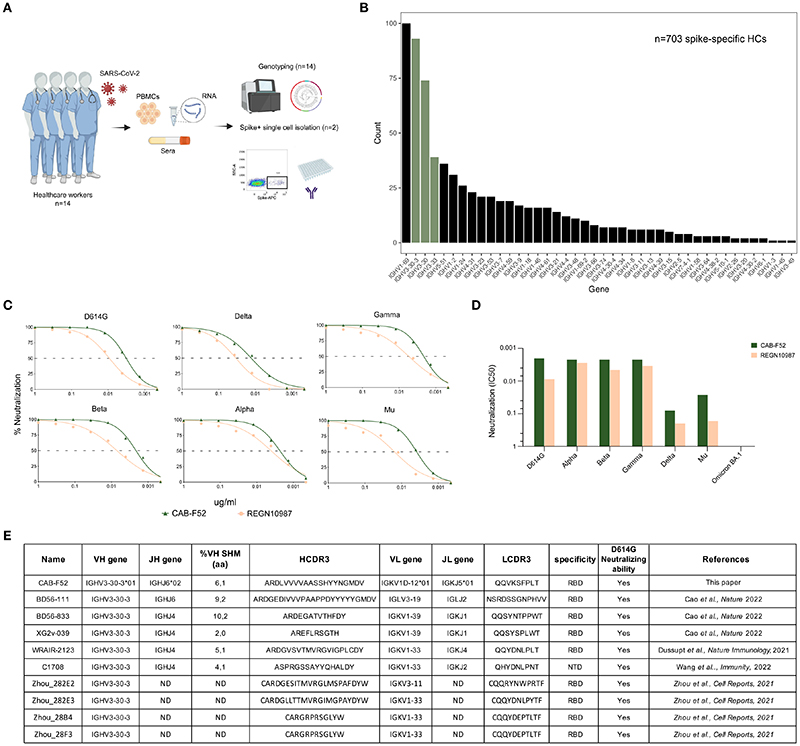
Spike HCs frequently use IGHV3-30 group of genes. **(A)** A schematic representation of the SARS-CoV-2 cohort consisting of 14 individuals. PBMCs, sera and RNA samples were isolated from each of these individuals. The genotyping of 14 study participants was done using the RNA sample. Two of the 14 individuals’ PBMCs were used to isolated S-specific HCs and subsequent antibody expression. **(B)** A bar plot showing the IGHV gene usage of S-specific HCs with IGHV3-30 group highlighted in olive green. **(C)** Titration curves showing percent neutralization of CAB-F52 and REGN10987 against VoCs. **(D)** IC50 neutralization values of CAB-F52 and REGN10987 against the VoCs. **(E)** A table showing the genetic properties, specificity, and neutralization activity of CAB-F52 and several IGHV3-30-3-using mAbs from the CoVAbDab.

We used a previously described cohort of healthcare workers (*n* = 14) (16), who tested positive for SARS-CoV-2 by RT-PCR in Spring 2020. As described, peripheral blood mononuclear cells (PBMCs) were collected seven months after the primary infection and all study participants (SPs) were individually genotyped for their IG V, D and J allele content using the germline gene inference tool IgDiscover (23). Single S-specific IgG memory B cells were sorted from two SPs and mAbs were isolated as described (Figure 1A) (16). A total of 703 S-specific HCs were obtained from the sorted cells. Examination of IGHV gene usage showed that 93 of the 703 S-specific sequences (13%) used the IGHV3-30-3 gene, making it the second most utilized IGHV gene in these individuals after IGHV1-69. IGHV3-30 and IGHV3-33 were the third and fourth most used genes in S responses, respectively (Figure 1B). Preferential IGHV gene usage was particularly evident for IGHV3-30-3, where the percentage of HCs using this gene was markedly higher in the S-specific response than in total IgG libraries from the same individual as we showed previously (16).

Amplification of antibody V(D)J regions from the S-specific B cells was performed by single-cell RT-PCR using previously described primers (30) for production of a panel of mAbs, as recently described (16). From this panel, we selected the IGHV3-30-3-using mAb, CAB-F52, for further analysis since this mAb displayed exceptional neutralization potency with an IC50 value of 0.005 against D614G. We tested the neutralization potency of CAB-F52 against several VoCs including Alpha, Beta, Gamma, Delta, Mu, and Omicron (BA.1) alongside a commercial IGHV3-30-3-using mAb, REGN10987 (31) and found that the potency of CAB-F52 was consistently higher when compared to that of REGN10987 against all VoCs except Omicron (BA.1) as demonstrated in the full dilution curves (Figure 1C) and by half maximal inhibitory concentration (IC50) values (Figure 1D). Several IGHV3-30-3-using neutralizing mAbs were also identified in other SARS-CoV-2 studies (13, 18, 32, 33), underscoring that such antibodies are frequently elicited following exposure to S (Figure 1E).

### CAB-F52 retains neutralization and breadth upon germline reversion

**Figure 2:**
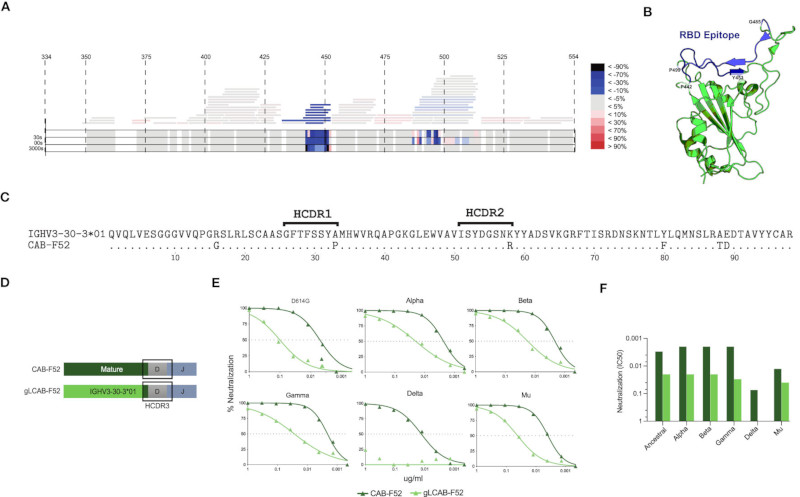
CAB-F52 targets RBD and retains neutralization activity upon germline reversion **(A)** Epitope mapping of CAB-F52 using HDX-MS. **(A)** A heat map showing the most probable (blue) and least probable (red) interaction sites of CAB-F52 on the RBD **(B)** A three-dimensional image of the targeted epitope **(C)** Amino acid sequence alignment of CAB-F52 VH region and germline allele IGHV3-30-3*01 allele. **(D)** Cartoon showing the gLCAB-F52 HC design **(E)** Titration curves showing percent neutralization of CAB-F52 and gLCAB-F52 against VoCs. **(F)** IC50 neutralization values of CAB-F52 and gLCAB-F52 against several VoCs.

To map the epitope specificity of CAB-F52, we generated a Fab version of the antibody and used hydrogen-deuterium exchange mass spectrometry (HDX-MS) to map the binding site on the ancestral RBD. The results demonstrated that the main interaction sites for CAB-F52 involved amino acid (aa) residues 450-480 and 485-500 of the RBD (Figure 2A). When modelling the epitope on the RBD structure, the binding mode overlapped that of SARS-CoV-2 neutralizing class 2 or 3 antibodies described by Barnes et al. (20) (Figure 2B). This likely explains why CAB-F52 is unable to neutralize Omicron BA.1, which harbors the T478K and G446S mutations that are known to affect binding by class 2 and class 3 antibodies, respectively (34). However, CAB-F52 retains neutralization capacity against Beta, demonstrating that it is insensitive to E484K.

B cells undergo affinity maturation of their recombined antigen receptors through the germinal center (GC)-mediated SHM process. To evaluate the contribution of the HC SHM for the neutralizing activity displayed by CAB-F52, the antibody was reverted to its germline configuration, keeping the mature HC complementarity determining region 3 (HCDR3) intact. An alignment of the CAB-F52 IGHV region and the germline IGHV3-30-30*01 sequence shows the positions in which the mature antibody had acquired SHM (Figure 2C). At the nucleotide level, the IGHV SHM level of CAB-F52 was 3.4%: a total of 6 aa substitutions, one of which was in the HCDR1 and one in the HCDR2. The aa substitutions in the HCs of CAB-F52 were reverted to the germline residues by replacing the VH-region with its assigned germline sequence, IGHV3-30-30*01 keeping the HCDR3 intact (Figure 2D). One SHM substitution in the HC J gene outside of the HCDR3 was also reverted to the germline state. The germline-reverted HC was paired with the mature CAB-F52 LC. Assessment of neutralization of the germline-reverted CAB-F52 (gLCAB-F52) compared to the mature mAb against the ancestral SARS-CoV-2 strain demonstrated that gLCAB-F52 retained its ability to neutralize the virus, though the potency was reduced by approximately ten-fold compared to the mature CAB-F52 (Figure 2E). gLCAB-F52 also retained neutralization breadth against several VoCs, except Delta as shown in the titration curves (Figure 2E) and IC50 values (Figure 2F). These results highlight the role of SHM for improving the potency of CAB-F52 such that it also neutralized the highly infectious Delta variant.

### Homozygous absence of IGHV3-30-3 affects the composition of the IgM repertoire

**Figure 3:**
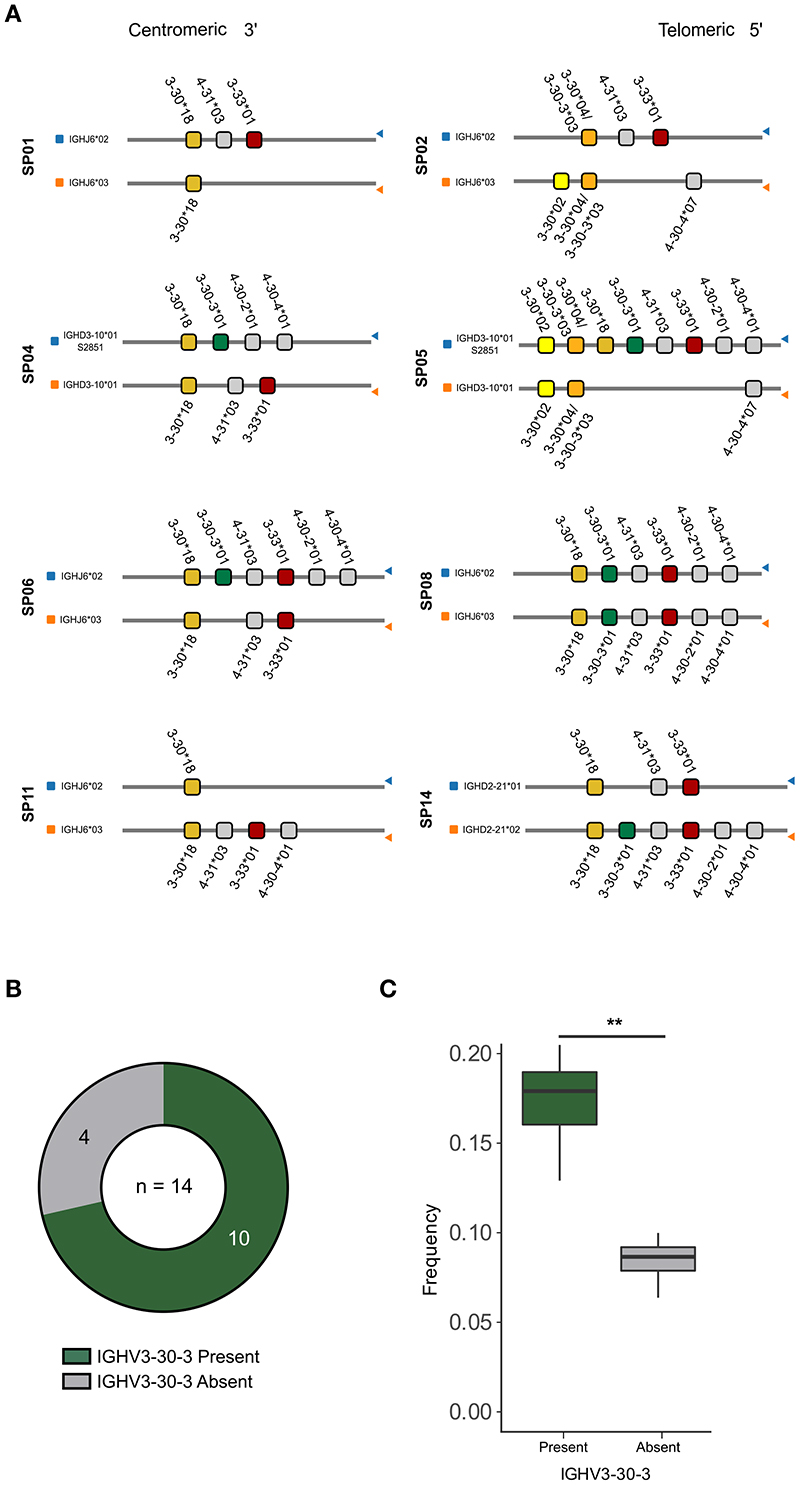
Haplotype and genotype analysis reveals variation and IGHV3-30-3 deletion **(A)** Illustration of IGHV3-30, IGHV3-30-3, IGHV3-33 alleles presence on each chromosome of the haplotypable study participants. **(B)** A pie chart showing frequencies of individuals with the IGHV3-30-3 gene present (green) or absent (gray). **(C)** Combined IGHV3-30/IGHV3-30-3 frequencies in IgM libraries of individuals with the IGHV3-30-3 present (green) or absent (gray). ** refers to the significant difference between the groups indicated by p value ≤ 0.01.

We next examined the germline-encoded variation in the IGHV3-30 family of genes in our study participants. We previously determined the IG V, D and J allele content in the 14 study participants (16) and here we employed the process of inferred haplotype analysis to determine which alleles are present on which chromosome. This process relies on the fact that VDJ recombination occurs locally within the set of V, D and J genes present on the maternally or paternally derived IGH locus and therefore, heterozygous IGHJ or IGHD alleles can be used as anchors to position IGHV alleles to either of the two chromosomes (35–37). We found that SP01, SP02, SP06, SP08, and SP11 were heterozygous for IGHJ (IGHJ6*02/IGHJ6*03), while SP14 was heterozygous for IGHD2-21 (IGHD2-21*01/IGHD2-21*02), and SP04 and SP05 were heterozygous for IGHD3-10 (IGHD3-10*01/IGHD3-10*01_S2851) (Figure 3A). Thus, we used these heterozygous IGHJ or IGHD alleles as anchors to haplotype eight of the 14 SPs. The inferred haplotype analysis demonstrated great inter-individual variability in the IGHV3-30 and IGHV3-30-3 allele content (Figures 3A, S1A). Several of the neighboring genes, IGHV4-30-2, IGHV4-30-4, and IGHV3-33, were also absent from these haplotypes, consistent with high structural variation in this genomic region (Figure 3A). Two cases, SP02 and SP05, were homozygous for IGHV3-30*04/IGHV3-30-3*03, which cannot be distinguished as they are identical the nucleotide level. To investigate which gene this sequence was likely to represent, we examined the total IGHV3-30*04/IGHV3-30-3*03 expression counts present on each chromosome in SP02, a donor who also lacked IGHV3-30-3*01 (Figure S1B). This revealed that there were more than twice as many IGHV3-30*04/IGHV3-30-3*03 read counts associated with the IGHJ6*02 chromosome compared to the IGHJ6*03 chromosome, suggesting that both IGHV3-30*04 and IGHV3-30-3*03 are present on the IGHJ6*02 chromosome in SP02 while only one of the genes is present on the IGHJ6*03 chromosome (Figure S1B). While we could not definitely confirm that this is the case, we concluded that the haplotype result of SP02 is consistent with a genotype containing both IGHV3-30*04 and IGHV3-30-3*03 on the IGHJ6*02 anchored chromosome. SP02 therefore likely contained one copy of the IGHV3-30-3 gene, while 4 of 14 donors lacked IGHV3-30-3 altogether (Figure 3B).

To understand the consequence of homozygous IGHV3-30-3 absence, we computed the combined frequencies of transcripts assigned to either IGHV3-30 or IGHV3-30-3 in the IgM libraries of each of the 14 SPs, having ensured that the sizes of the IgM libraries were comparable (Figure S1C). The results of the analysis showed that the individuals who lacked IGHV3-30-3 had distinctly fewer IGHV3-30 and IGHV3-30-3 IgM sequences (Figures 3C, S1D), which may impact their capacity to make CAB-F52-like antibodies and other responses involving these genes.

### IGHV3-30 group gene variation in an expanded study group and evaluation of functional redundancy

**Figure 4:**
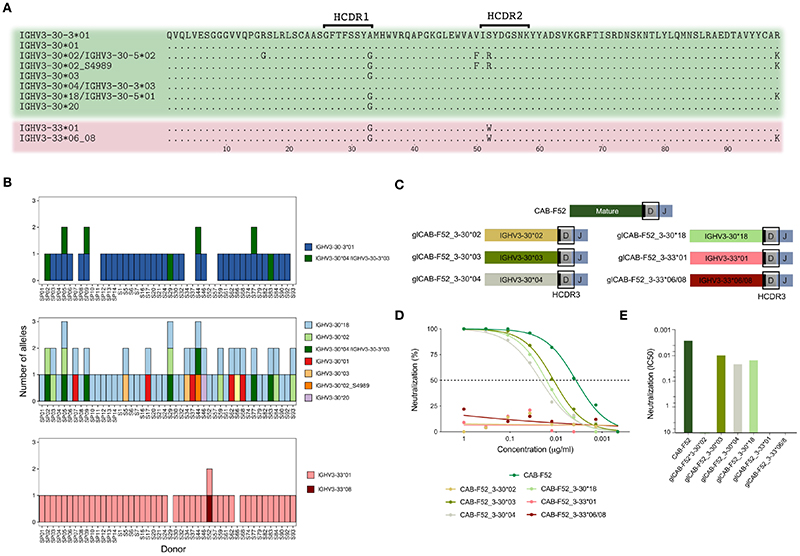
IGHV3-30 group of genes are highly variable and can impact CAB-F52 function. **(A)** Alignment of all the amino acid sequences of IGHV3-30, IGHV3-30-3 and IGHV3-33 alleles present in the extended group of 46 cases. **(B)** Allelic content in the 46 cases: IGHV3-30 (top graph), IGHV3-30-3 (middle graph) and IGHV3-33 (lower graph). The IGHV3-30*04/IGHV3-30-3*03 (dark green) is shown in both the IGHV3-30-3 and IGHV3-30 plots since they cannot be distinguished **(C)** Cartoons showing the design of the germline-reverted CAB-F52 HC variants. **(D)** Titration curves showing percentage neutralization of the allele-swapped CAB-F52 mAb variants. **(E)** IC50 neutralization values of the allele-swapped CAB-F52 mAb variants.

IGHV3-30, IGHV3-30-3 and IGHV3-33 are closely related at both nucleotide and amino acid levels, ranging from one to five polymorphic coding positions (Figure 4A). As mentioned above, IGHV3-30*04 and IGHV3-30-3*03 have identical nucleotide sequences and cannot easily be distinguished from one another. Here, to indicate this ambiguity, we labeled these sequences as IGHV3-30*04/IGHV3-30-3*03.

To expand the analysis of the IGHV3-30 group germline variation, we analyzed publicly available IgM repertoire data from an additional cohort of 32 individuals from an independent study (24). Examination of the combined 46 cases revealed that 9 individuals (19.5%) lacked IGHV3-30-3*01 on both chromosomes (Figure 4B). In addition to these 9 cases, 3 more individuals had the ambiguous IGHV3-30*04/IGHV3-30-3*03 sequence. We also examined the IGHV3-30 and IGHV3-33 allele content in the 46 cases. The IGHV3-30 allele content varied greatly between the cases (Figure 4B). IGHV3-30*18 was the most common IGHV3-30 allele and was found in all but one case (Figure 4B). The IGHV3-33 gene was less variable, with the IGHV3-33*01 allele present in 44 cases and homozygous absence of the gene only observed in two cases.

To test whether IGHV3-30-3 can be functionally replaced by other genes of the IGHV3-30 group we designed mutated versions of CAB-F52 HCs, replacing its VH-region with alleles IGHV3-30*02, IGHV3-30*03, IGHV3-30*04, IGHV3-30*18, IGHV3-33*01 and IGHV3-33*06/08 allele germline sequences and then expressed them as mAbs using mature CAB-F52 LCs (Figure 4C). All the IGHV3-30 allele-swapped mAbs retained neutralization except for the IGHV3-30*02 demonstrating the functional similarities between the IGHV3-30 and many IGHV3-30-3 alleles. On the other hand, replacing the CAB-F52 VH-region with the IGHV3-33 alleles resulted in a complete loss of neutralization capability illustrating the functional impact of even a single germline-encoded amino acid difference (Figures 4D, E).

## Discussion

Inter-individual variation in immune genes is a characteristic of mammals. This leads to heterogeneity in responses at individual levels that benefits the population, for example during pandemics. The most variable of the three loci that encode the IG genes is the IGH locus, present at the telomeric end of chromosome 14. This locus is characterized by extensive structural variation involving frequent segmental deletions, insertions and duplications of a subset of IGH genes, suggesting higher genomic instability in these locations (22–25). Regions within the IGH locus displaying especially high variability include the region spanning the IGHV3-30, IGHV3-30-3, and IGHV3-33 genes, resulting in several haplotypes that vary in length by over 50 kb (38–42). Furthermore, the IGHV3-30 gene displays an unusually high level of allelic variation, with additional novel alleles discovered as the IG germline allele content in additional individuals are defined.

IGHV3-30, IGHV3-30-3 and IGHV3-33 are some of the most highly utilized genes in the naive B cell repertoire. Since the Covid-19 pandemic, several independent studies that have characterized the S-specific antibody response indicate preferential usage of IGHV3-30 and IGHV3-30-3 in addition to several other genes including IGHV1-69, IGHV3-9, IGHV3-53, and IGHV5-51 (15–18). Many mAbs isolated from early time points following infection display low levels of SHM yet moderate-strong neutralizing activity, suggesting that their naive, germline-encoded states have sufficient affinity to the targeted neutralizing epitopes (12, 17). Since some of these antibodies use IGHV genes that are highly variable between individuals, such as IGHV1-60, IGHV3-30, or IGHV3-30-3, it follows that differences in IG genotypes may impact the early immune control of primary SARS-CoV-2 infection.

The fact that an individual’s antigen-specific antibody repertoire can be influenced by IGHV germline gene composition and usage underscores the importance of personalized IG genotyping for a comprehensive understanding of B cell responses, as demonstrated by recent studies (16).

In the current study, we isolated an IGHV3-30-3 using mAb that was ultrapotent and showed breadth against VOCs, adding to the list of several additional IGHV3-30-3-using neutralizing antibodies reported in the literature. The functional characterization of CAB-F52 revealed its epitope on the spike RBD to be overlapping with the ACE-2-binding region and with that of class 2 and 3 antibodies (20). HC germline-reversion of CAB-F52 reduced the neutralizing activity compared to the mature antibody, but the germline-reverted antibody retained considerable potency and breadth. Due to the high sequence homology between IGHV3-30 and IGHV3-30-3, we asked if CAB-F52 could have used IGHV3-30, which is almost always present in individuals, sometimes in more than one copy per chromosome.

Studies have shown that gene copy numbers can influence IGHV gene expression in the repertoire (43, 44). Although homozygous absence of IGHV3-30-3 may be functionally replaced by IGHV3-30, our analysis of the combined frequencies of IGHV3-30 and IGHV3-30-3 sequences in IgM repertoires revealed that genomic absence of IGHV3-30-3 can greatly decrease the total number of IgM B cells encoding these highly similar V genes and may, therefore negatively influence responses that rely on IGHV3-30 group gene usage. In contrast, if several different IGHV3-30 genes and alleles show functional redundancy, a higher allelic copy number will result in some individuals showing a higher early response, particularly in situations where germline-encoded antibodies have high affinity to their target epitope in the absence of SHM.

Overall, the biological consequence of variation in IGHV genes remain incompletely understood. While we observed redundant function between several IGHV3-30 and IGHV3-30-3 alleles in the context of CAB-F52, despite their overall similarity, the IGHV3-33 alleles were sufficiently different that they could not substitute for the native IGHV3-30-3 germline gene. We observe that functional redundancy existing between similar V genes or alleles can, however, be abrogated by even single amino acid differences. This has important implications for the high level of IGHV allelic variation found in the human population. The requirements for specific germline gene and allele usage are expected to depend greatly on the specific binding modes, which vary greatly between different antibodies. Further cohort studies that investigate the impact of IG germline gene variation to biological outcomes in the context of infections and vaccinations will offer further insight. Such studies will be particularly important for vaccine design aimed at eliciting specific classes of antibodies, so-called germline targeting vaccine strategies (45), where knowledge of IG allele frequencies in the targeted population is of particular importance.

### Limitations

A limitation of our study is sample size. The germline reversion and allele swapping experiments were only done in the context of a single mAb, CAB-F52, and the functional redundancy of the IGHV3-30 and IGHV3-30-3 alleles may not be true for other antibodies. Furthermore, we did not perform germline reversions of the CAB-F52 light chain V gene since the focus of the paper was on germline heavy chain V genes. For the genotyping studies, we addressed the small sample size by extending our cohort with the individuals from Gidoni et al. Nevertheless, further studies that couple genetic and functional analyses in larger cohorts are required to better understand functional consequences of germline IG gene variation.

## Supplementary Material

Supplementary Material

Supplementary Table 1

## Data Availability

All data were collected as previously described (16). The HC and LC sequences of CAB-F52 and the IgM repertoire data from the 14 donors are available at GenBank under the accession numbers ON086926 and ON086941. Repertoire data is available from SciLifeLab at: http://doi.org/10.17044/scilifelab.19317512. The HDX data have been deposited to the ProteomeXchange Consortium via the PRIDE (29) partner repository with the dataset identifier PXD031945. The IgDiscover software can be found at http://docs.igdiscover.se/en/stable/. The plot allele module and the rep-seq analysis tools can be found under IgDiscover v0.12.4.dev22+g0bc3365.
