# Supplementary material for: Frequent use of IGHV3-30-3 in SARS-CoV-2 neutralizing antibody responses
Source: Front Virol. Author manuscript; Available in PMC 2023 Apr 10. (PMC7614418; doi:10.3389/fviro.2023.1128253)
Supplement: Supplementary Material [file EMS173346-supplement-Supplementary_Material.PDF]

A

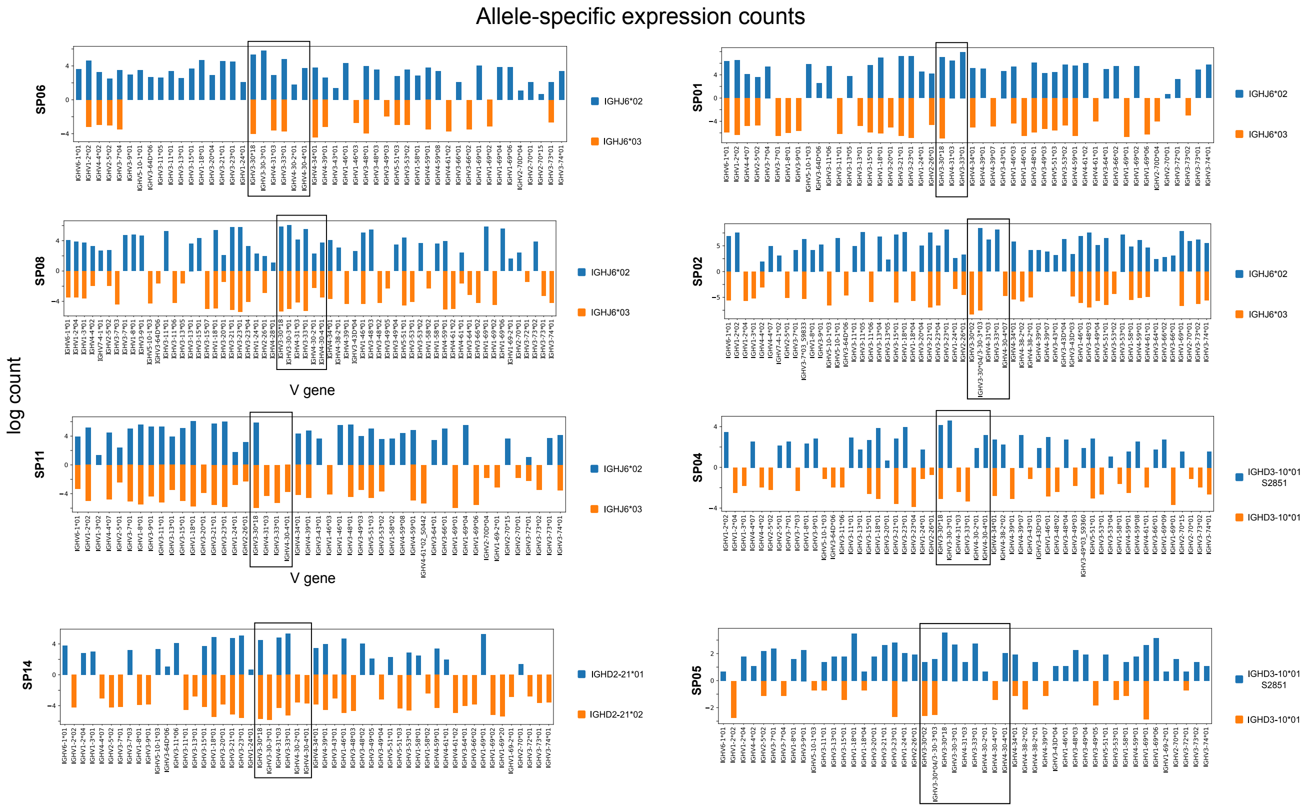

B

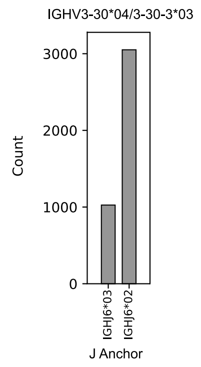

C

ID	Total reads	Merged reads	post FAD
SP01	903862	849704	10731
SP02	966158	829456	7689
SP03	806012	778947	20143
SP04	911283	871532	23623
SP05	969970	890207	11843
SP06	917921	773708	6220
SP07	898039	793103	10627
SP08	883497	841771	10028
SP09	774086	749100	19192
SP10	922305	891937	15185
SP11	732509	713829	16546
SP12	872869	856214	23509
SP13	1130804	1093718	11814
SP14	1316922	1302749	86798

D

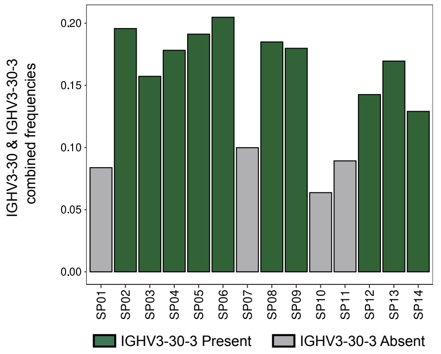

Figure S1. A. Bar graphs show the haplotype plots of the SPs with heterozygous IGHJ or IGHD alleles as anchors. Orange and blue bars indicate each chromosome sequence counts on the y-axis. The x-axis has the alleles shown in chromosomal order. **B.** The bar graph shows the total chromosomal counts of IGHV3-30*04/IGHV3-30-3*03 in SP02. **C.** Table showing the IgM library sizes of the SPs in terms of total and merged reads. post FAD represents the final number of sequences used to do analyses. **D.** The bar graph represents the IGHV3-30 and IGHV3-30-3 combined IgM frequencies of 14 SPs.
